# Pleiotropic Effects of Simvastatin on the Regulation of Potassium Channels in Monocytes

**DOI:** 10.3389/fphar.2020.00101

**Published:** 2020-02-21

**Authors:** Shaoping Wang, Yuhua Ran, Xuejun Chen, Chungang Li, Shujuan Cheng, Jinghua Liu

**Affiliations:** ^1^ Department of Cardiology, Beijing Anzhen Hospital, Capital Medical University, Beijing Institute of Heart Lung and Blood Vessel Diseases, Beijing, China; ^2^ Department of New Drug Evaluation, State Key Laboratory of Toxicology Medical Courtermeasures, Institute of Pharmacology and Toxicology, Beijing, China; ^3^ Research Institute of Chemical Defense, Beijing, China; ^4^ No. 926 Hospital, Joint Logistics Support, Force of PLA, Yunan, China

**Keywords:** statin, potassium channel, peripheral monocyte, cell migration, coronary artery disease

## Abstract

**Purpose:**

The underlying mechanism of pleiotropic effects of statins on atherosclerosis is still unclear. Kv1.3 and KCa3.1 are two potassium channels that might be involved in monocyte migration and atherosclerosis formation. The aim of this study was to investigate the effect of simvastatin on the Kv1.3 and KCa3.1 in monocyte.

**Methods and Results:**

In human monocytic THP-1 cells, simvastatin significantly inhibited Kv1.3 mRNA and protein expression by real-time quantitative PCR analysis and western blotting. However, simvastatin had no effects on KCa3.1 mRNA and protein expression. By whole-cell patch clamp, simvastatin (10 μM) remarkably inhibited the current intensity of Kv1.3, but had no effect on KCa3.1. Simvastatin (10 μM) treatment significantly reduced the monocyte chemoattractant protein 1 (MCP-1)-induced monocyte migration. This inhibition was only partially reversed by mevalonate (1mM). In human peripheral blood mononuclear cells (PBMCs), both Kv1.3 and KCa3.1 mRNA expression were increased in patients with coronary artery diseases (CAD) (n = 20) compared to healthy controls (n = 22). However, simvastatin (40 mg per day) significantly inhibited the Kv1.3 but not KCa3.1 mRNA expression after 1 month and 3 months therapy in CAD patients.

**Conclusion:**

Our data suggested Kv1.3 in monocytes was a potential molecular target of the pleiotropic effects of statins. KCa3.1 might be another marker of CAD, but not associated with statins treatment.

## Introduction

Coronary artery disease (CAD) engenders serious threats to human health and has become an important public health problem worldwide (Benjamin et al., 2017; Chen et al., 2017). The migration of peripheral blood monocyte cells (PBMCs) as well as the accumulation in arteries and, subsequently, the release of large amounts of inflammatory factors by macrophages are key steps in the early formation of atherosclerosis (Rader and Daugherty, 2008).

Statins are one of the most effective drugs developed for the treatment of CAD and are widely used clinically as lipid-lowering drugs. A series of large-scale clinical trials have shown that statins can significantly reduce the incidence and mortality of cardiovascular events and play an important role in primary (Ridker et al., 2008) and secondary prevention (Stone et al., 2014) of CAD. It has been clarified that the long-term benefits of statins are far greater than the lipid-lowering effect alone. The pleiotropic effects of statins may include improving vascular endothelial function, stabilizing plaques, improving myocardial remodeling, and reducing neuroendocrine activation, but the specific mechanisms underlying these effects are still unclear (Athyros et al., 2009). It has been reported that statins were associated with the reduction of the expression of adhesion molecules and pro-inflammatory cytokines in human monocytes (Han et al., 2005; Walter et al., 2010). Monocytes may be one of the targets of statins, and the effects of statins on monocytes may be the possible mechanism underlying early intervention for atherosclerosis.

Kv1.3 and KCa3.1 are two potassium channels that are consistently demonstrated to be associated with atherosclerosis (Kohler et al., 2003; Toyama et al., 2008; Zhu et al., 2015; Wu et al., 2015; Kan et al., 2016). Margatoxin (MgTX) can specifically block the Kv1.3 potassium current, and Tram-34 is a KCa3.1-specific blocking agent. Studies (Gendelman et al., 2009; Schilling and Eder, 2009) found that these two potassium currents in monocytes were enhanced by monocyte chemoattractant protein 1 (MCP-1), whereas specific blockade of Kv1.3 and/or KCa3.1 completely blocks the migration of MCP-1-induced cells. These two potassium channels therefore may play an important role in regulating the migratory function of monocytes. Recently, lovastatin (30 µM) was reported to exert immunomodulatory properties through the mechanism of blocking Kv1.3 channel in human T cells (Zhao et al., 2015). Simvastatin (10 uM) was also found to be an inhibitor of Kv1.3 in murine thymocytes (Kazama et al., 2014). However, its pathophysiology and clinical significance has not been clarified.

Therefore, we hypothesized that there was an increased expression of potassium channels in the PBMCs of CAD patients. The statin simvastatin can inhibit expression of potassium channels in monocytes from patients with CAD, inhibiting the migration of monocytes to exert its potential effect of anti-atherosclerosis. The aim of this study was to verify the potential of potassium channels in PBMCs as molecular markers of CAD and to elucidate the possible mechanisms underlying the pleiotropic effects of statins.

## Methods and Materials

### Ethics Statement

This study was performed in strict accordance with the recommendations of the National Institute of Health Guide for the Care and Use of Laboratory Animals (NIH Publications No.80-23, revised 1996), and all clinical investigation must have been conducted according to the principles expressed in the Declaration of Helsinki. The protocol was approved by the Ethics Committee of Beijing Anzhen Hospital, Capital Medical University.

### Study Population

A total of 42 patients presenting with chest discomfort were included from Beijing Anzhen Hospital. Each patient received coronary angiography. A total of 22 patients were diagnosed with coronary artery disease (CAD group), defined as a stenosis ≥50% in diameter in the left main or at least one major coronary artery. Another 20 patients (control group) had smooth coronary angiography. Patients who were diagnosed with acute myocardial infarction were excluded. To clarify the effect of simvastatin on the expression of Kv1.3 and KCa3.1, patients who had undergone statin therapy before hospitalization were also excluded. Peripheral blood samples were collected upon hospitalization (0 month) as well as 1 month and 3 months after simvastatin therapy of 40 mg per day in patients with CAD during the follow-up. 5 patients missed the follow-up at 3 months.

### Isolation and Culture of PBMCs

Peripheral blood obtained from CAD patients and control group was anticoagulated with heparin. PBMCs were separated on a Ficoll-Hypaque (F/H) density gradient (Lymphoprep, Nyegaard and Co., Oslo, Norway). Lymphocytes (PBLs) were obtained after adherence of PBMCs to plastic flasks for 1 h at 37°C. Adherent cells were obtained by scraping the flasks with a rubber spatula after carefully rinsing out non-adherent cells with three washes of PBS; these preparations contained >95% CD14+ monocytes. In several experiments, monocyte-free PBL preparations were obtained after two adherence cycles on plastic, sensitization with the IgM Ab B52.1 (anti-CD14) (ab182032, Cambridge, UK), elimination of agglutinated cells by filtration through nylon wool, and depletion of the remaining antibody-sensitized CD14^+^ monocytes by indirect resetting with anti-globulin-coated sheep erythrocytes and F/H gradient separation. These PBL preparations contained <1% (usually 0.1–0.5%) monocytes, as determined for each preparation by nonspecific esterase staining.

### Cell Migration Assay

Migration of THP-1 cells (human monocytic leukemia) was assessed with Boyden chambers using Transwell^®^ inserts (Corning Life Sciences, Oneonta, NY, USA) separated by a polycarbonate membrane with an 8-μm pore opening placed within 24-well plates. A 300 μl suspension of serum-starved RMLEC cells at a concentration of 2 × 10^5^ cells/ml of serum-free medium was seeded in the upper chamber in the presence of either PGE2, PGE1OH, or C3L5-CM, while the lower chamber had 2% charcoal-dextran-stripped FBS-containing medium (free of PGE2). The assembled chambers were then incubated for 24 h. After incubation, the cells from the top of the membrane were wiped off with cotton swabs, whereas the migrated cells (from the bottom of the membrane) were fixed with cold methanol, stained with eosin/thiazine, and washed with distilled water. The membranes were then dried, cut with a surgical blade, and fixed with mounting medium on a glass slide. Direct microscopic counting at 40 × magnification (Leica DFC 295, Leica Microsystems, Germany) of cells that migrated to the lower side of the membrane was performed, and a mean value for each sample was calculated.

### RNA Extraction and Real-Time qPCR for Kv1.3 and KCa3.1 Expression

Total RNA was extracted from PBMCs and THP-1 cells after specific treatments as detailed in the results section using an RNeasy Mini Kit. Respective cDNA was synthesized using a qScript™ cDNA Synthesis Kit, and real-time quantitative PCR analysis was performed with a BioRad thermocycler using PerfeCTa^®^ Green SuperMix; the data were analyzed using CFX Manager™ software for Rattus norvegicus β-actin, Kv1.3, and KCa3.1 gene expression. To determine the relative gene expression levels, the comparative threshold cycle method (ΔCt) was used (Livak and Schmittgen, 2001). The final mRNA levels were normalized according to their Ct values from the standard curves and expressed relative to the β-actin level. The following primer pairs were used: β-actin forward (5-tag gtt ttg tca aag aaa gg-3), reverse (5-tag gtt ttg tca aag aaa gg-3); Kv1.3 forward (5-tac tac gcc tga gtt tct ga-3), reverse (5-ggt gta gta gga gag gtt gg-3); KCa3.1 forward (5-ctt gaa aaa ctg ttg cca ca-3), reverse (5-aca aga gaa aaa cct cag ct-3). The respective amplicons formed were verified by DNA agarose gel analysis in conjunction with a standard DNA marker.

### Electrophysiology Determination

Whole-cell patch clamp recording was used in electrophysiology studies. The resistances of pipettes filled with pipette solutions ranged from 2 to 6 MΩ; before the pipette touched the cell, the liquid-junction potentials were compensated. Series resistance was compensated by approximately 75% ~ 90%. The data were acquired with an Axopatch 200B (Axon Instruments, Foster City, CA, USA). Membrane currents were low-pass filtered at 2 kHz. pClamp 9.2 software (Axon Instruments) was used for data analysis. The percent inhibition of the current was defined as [(I_control_ – I_simvastatin_)/I_control_] x 100%. The concentration of simvastatin required for 50% inhibition of the current (IC50) was determined by fitting the data with a Boltzmann equation. The composition of Tyrode’s solution was (in mmol/L): 140 NaCl, 4.5 KCl, 1.0 MgCl_2_, 2.0 CaCl_2_, 10 Glucose, 10.0 HEPES, pH 7.4. The pipette solution for Kv1.3 currents recording containing (in mM): 100 K^+^ aspartate, 40 KCl, 10 HEPES, 10 EGTA, and 1 MgCl_2_, pH7.2. The pipette solution for KCa3.1 currents recording containing (in mM): 100 K^+^ aspartate, 40 KCl, 1 MgCl_2_, 10 HEPES, 5 EGTA, and 4.4 CaCl_2_, pH7.2.

### Western Blotting

PBMCs and THP-1 cells were washed with ice-cold DPBS (including 10 nM NaF and 1 mM Na_3_VO_4_) and lysed in M-PER^®^ lysis buffer supplemented with a HALT™ protease inhibitor cocktail, 10 mM NaF and 1 mM Na_3_VO_4_. PBMCs were isolated as mentioned in *Isolation and culture of PBMCs*. Then, cell lysates were centrifuged, and supernatant protein was quantified using the BCA protein assay kit. Equal amounts of protein (25 μg) were separated on 12% SDS-PAGE gels and transferred to a PVDF membrane. Membranes were blocked in 5% non-fat milk in TBS (20 mM Tris-base, 0.14 M NaCl, pH 7.8) with 0.05% Tween-20 for one hour at room temperature and probed with respective primary antibodies for Kv1.3 and KCa3.1 (1:1000) at 4°C, overnight. The membranes were washed with TBS with 0.05% Tween-20 and incubated in HRP-conjugated rabbit or mouse secondary antibodies (1:5000) for one hour at room temperature. The specific band were detected and analyzed *via* Gel Pro Analyzer (Media Cybernetics, Bethesda, USA).

### Statistical Analysis

Data are presented as mean ± standard error of mean (SEM). Statistical analysis among three groups was performed by one-way ANOVA followed by LSD comparison test. Comparison of two groups was done parametrically (Student’s *t* test) or nonparametrically (Mann-Whitney rank sum test) according to the results of preliminary tests for within-group normality and equality of variances. All statistical analyses were based on two-tailed tests. Values of *P* < 0.05 were considered statistically significant.

## Results

### Effects of Simvastatin on the mRNA Expression of Kv1.3 and KCa3.1 in THP-1 Cells

**Figure 1:**
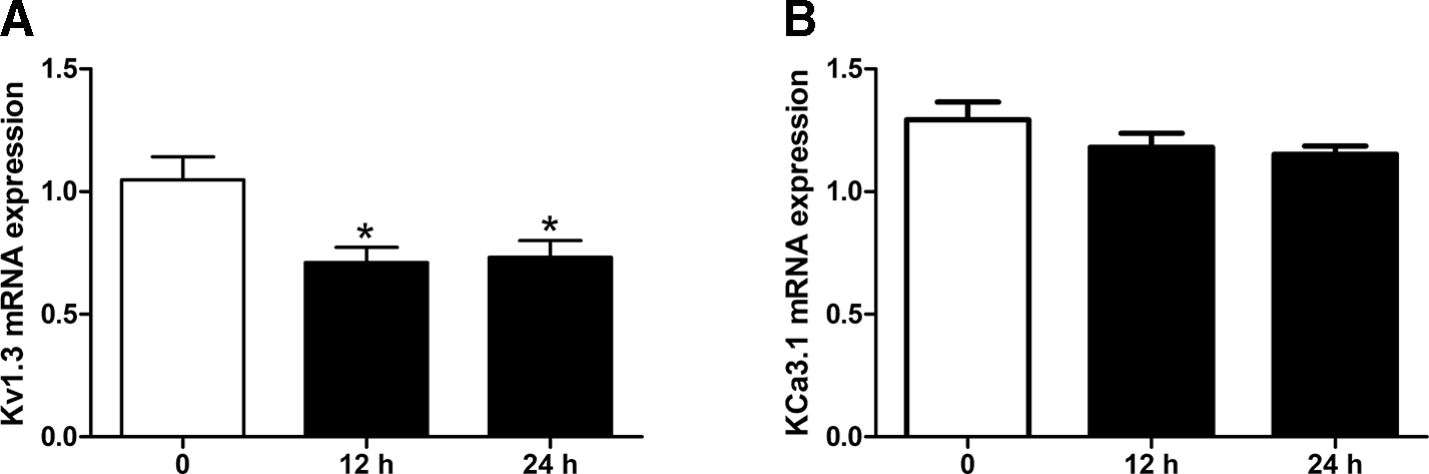
Effects of Simvastatin on mRNA expression of Kv1.3 and KCa3.1, respectively, in monocytic THP-1 cells determined by quantitative real-time PCR. **(A)** Simvastatin treatment remarkably inhibited the mRNA expression level of Kv1.3 in monocyte THP-1 cells. **(B)**. Simvastatin treatment had no influence on the mRNA expression level of KCa3.1 in monocyte THP-1 cells. *P < 0.05 *vs.* controls n = 12.

The effects of simvastatin (10 μM) treatment on the mRNA expression levels of Kv1.3 and KCa3.1 in THP-1 cells were measured using quantitative real-time PCR. Simvastatin (10 μM) was pre-incubated with THP-1 cells for 12 hours or 24 hours before extraction. Simvastatin treatment significantly reduced the mRNA expression level of Kv1.3 compared with the controls, *P < 0.01 (**Figure 1A**). There was no significant difference between 12 hours and 24 hours pre-incubation of simvastatin. By contrast, the mRNA expression level of KCa3.1 had no significant changes among simvastatin and control groups (**Figure 1B**); thus, simvastatin had no influence on the mRNA expression level of KCa3.1 in THP-1 cells.

### Effects of Simvastatin on Kv1.3 and KCa3.1 Protein Expression in PBMCs and THP-1 Cells

**Figure 2:**
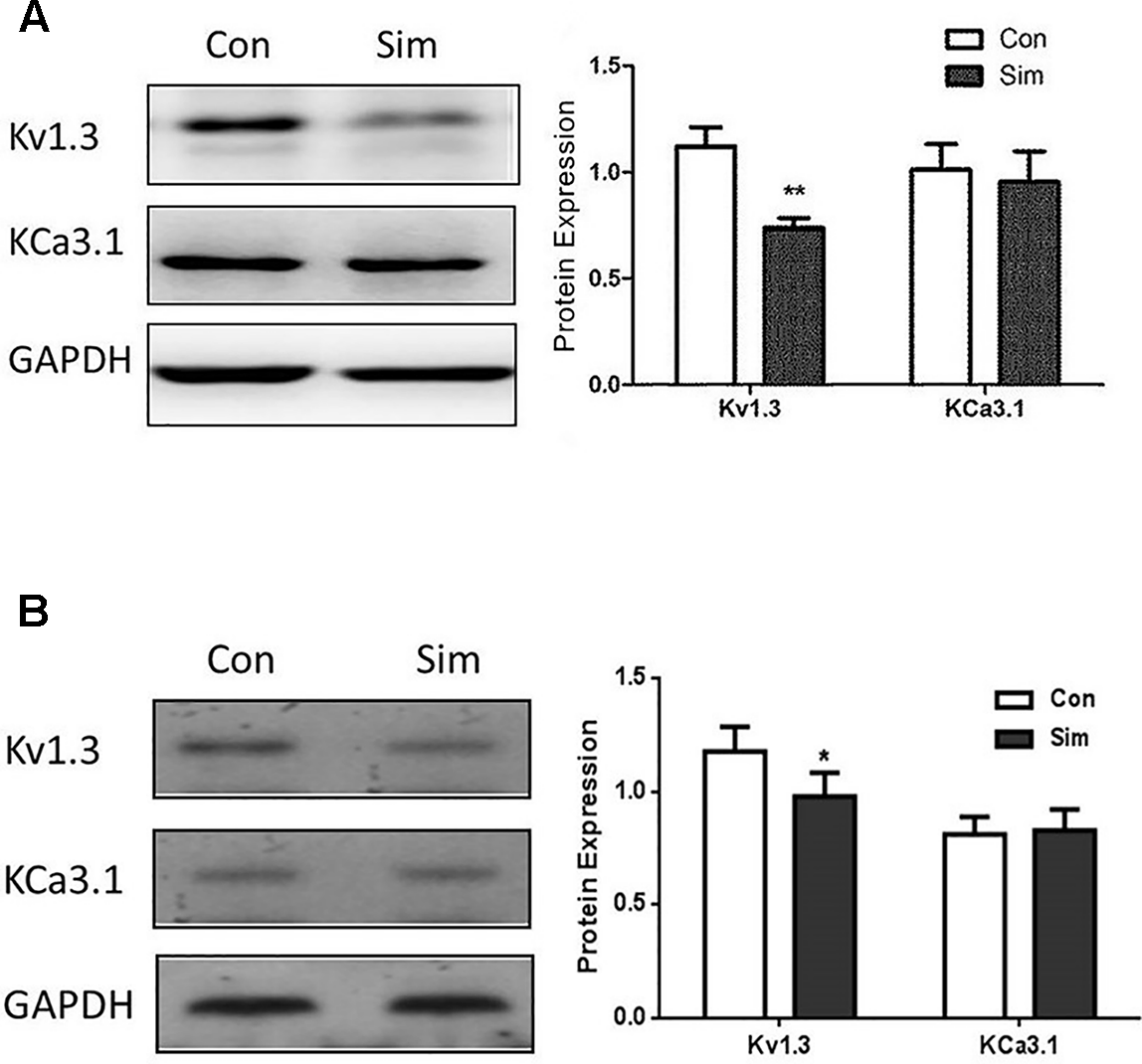
Protein expression levels of Kv1.3 and KCa3.1 in THP-1 cells **(A)** and PBMCs from CAD patients **(B)** were measured by western blot, respectively. **(A)** Representative western blot images of Kv1.3 and KCa3.1 levels in normal (control) and simvastatin treatment groups in THP-1 cells. The results were quantified, and represent the mean ± SEM, compared to controls (n = 4, **P < 0.01). **(B)** Representative western blot images of Kv1.3 and KCa3.1 levels in normal (control) and simvastatin treatment groups in PBMCs from CAD patients. The results were quantified, and represent the mean ± SEM, compared to controls (n = 4, *P < 0.05).

The protein expression levels of Kv1.3 and KCa3.1 were also investigated in THP-1 cells by Western blotting. Both ion channels were expressed in THP-1 cells, which conformed to the mRNA expression pattern in THP-1 cells. After simvastatin treatment for 24 hours, the protein expression of Kv1.3 was remarkably decreased in the THP-1 cell line *vs.* the normal control, **P < 0.01. In contrast, the protein level of KCa3.1 showed no significant change (**Figure 2A**). This finding suggests that only (Kv1.3 not KCa3.1) is involved in the regulatory mechanism of simvastatin, and the protein expression levels of Kv1.3 and KCa3.1 were investigated in PBMCs from CAD patients by western blotting. Both ion channels were expressed in PBMCs, which conformed to the mRNA expression pattern above. After Simvastatin treatment for 24 hours, the protein expression of Kv1.3 was decreased in the PBMCs *vs.* the normal control, *P < 0.05. In contrast, the protein level of KCa3.1 showed no significant change (**Figure 2B**). The data were consistent with the THP-1 cell (**Figure 2A**).

### Effects of Simvastatin on the Currents of Kv1.3 and KCa3.1 Channels in THP-1 Cells

**Figure 3:**
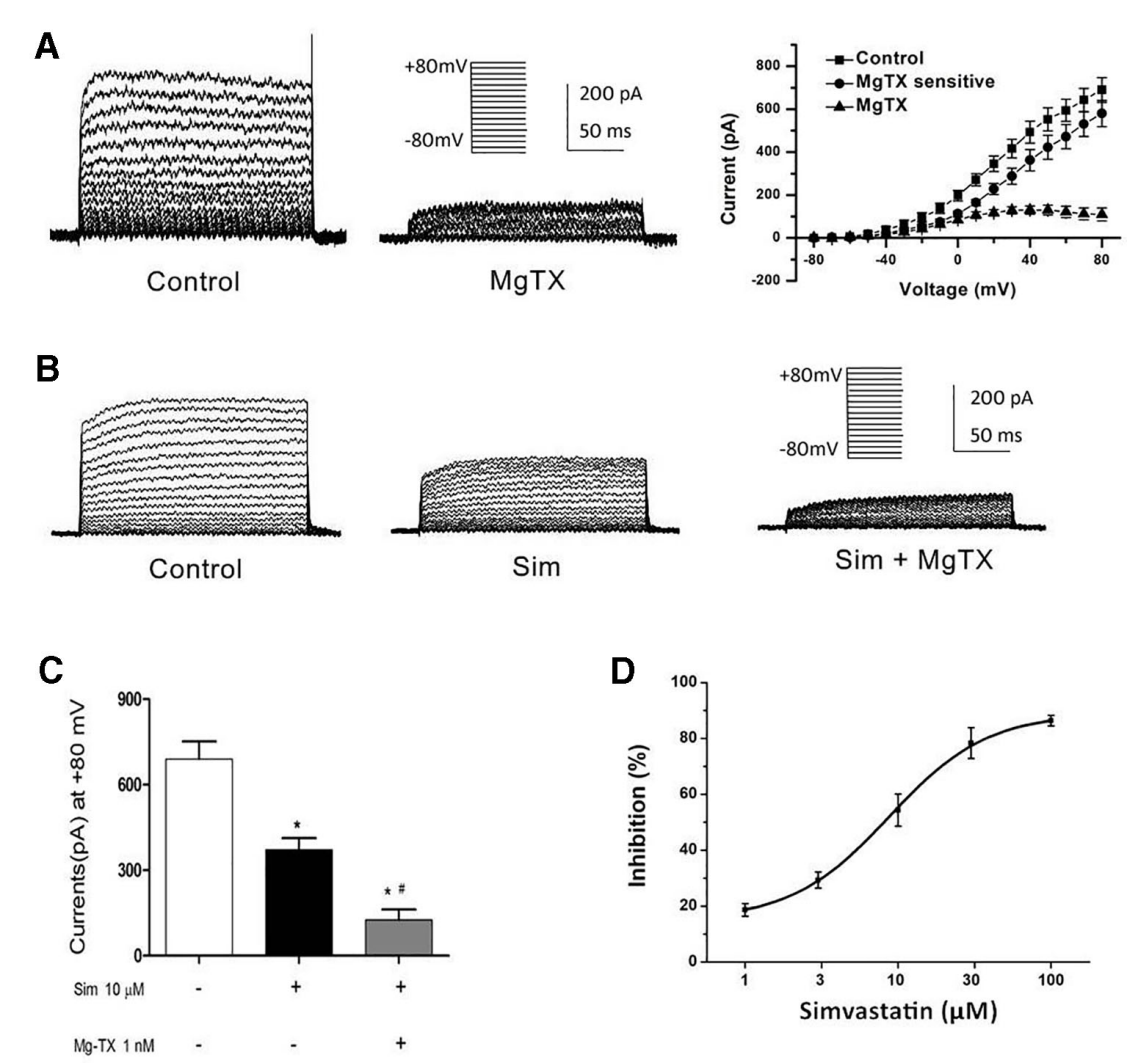
Effects of simvastatin on Kv1.3 current intensity change in THP-1 cells measured by whole-cell clamp patch. **(A)** Kv1.3 current was detected with a series of test potentials of 300 ms duration from −80mV to +80mV in 10 mV increments from a holding potential of −70mV. The currents were blocked by 1 μM MgTX. **(B)** Simvastatin treatment significantly inhibited the current intensity of Kv1.3. **(C)** The 10 μM simvastatin treatment remarkably inhibited the current intensity of Kv1.3 *vs.* control, *P < 0.05 *vs.* controls, and the addition of 1 nM MgTX further inhibited the current intensity compared with the simvastatin treatment alone, ^#^ *P* < 0.05 *vs.* inhibitor treatment, when the voltage was set at +80 mV, n = 4 for each group. **(D)** The does-response data of the Kv1.3 current at +40 mV was summarized (n = 4 for each concentration) and fitted with Boltzmann equation. The IC_50_ was calculated to be 8.75 ± 1.25 μM.

**Figure 4:**
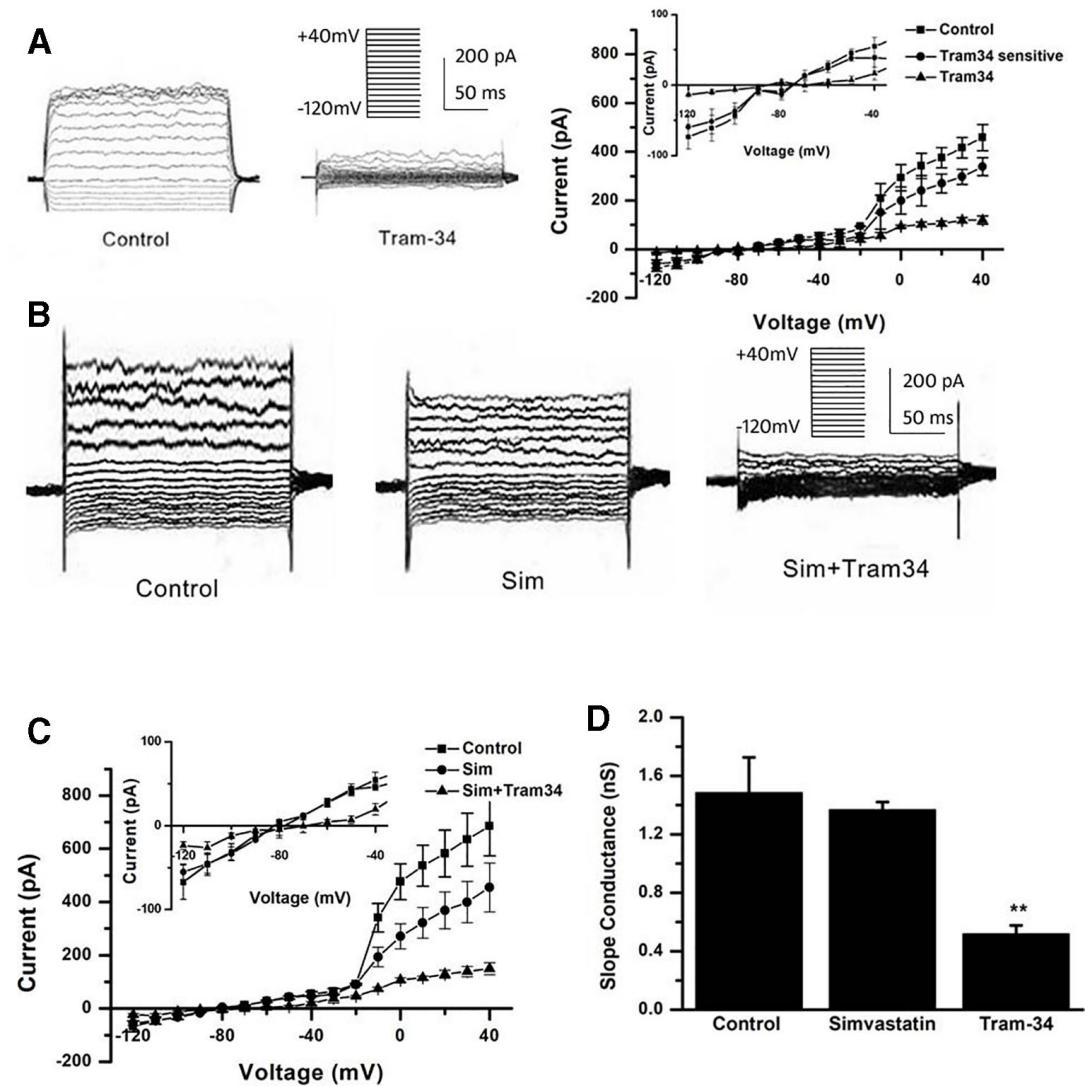
Effects of simvastatin on KCa3.1 current intensity change in THP-1 cells measured by whole-cell clamp patch. **(A)** The THP-1 cells were held at −40mV and received the test potentials from −120 to +40 mV; the KCa3.1 currents were detected by using an electrode with 1 µM free Ca^2+^. The currents were blocked by 1 μM Tram-34. **(B)** The representative current traces of KCa3.1. Simvastatin treatment had no significant effect on the current intensity of KCa3.1. **(C)** Average current density-voltage relationships before and after application of 10 µM simvastatin and 1 µM Tram-34 was indicated (n = 4 for each group). **(D)** The slope conductance between −120 mV and −40 mV was measured by fitting the curve with linear equation. Data was summarized from 4 THP-1 cells, **P < 0.01, *vs.* normal controls.

**Figure 5:**
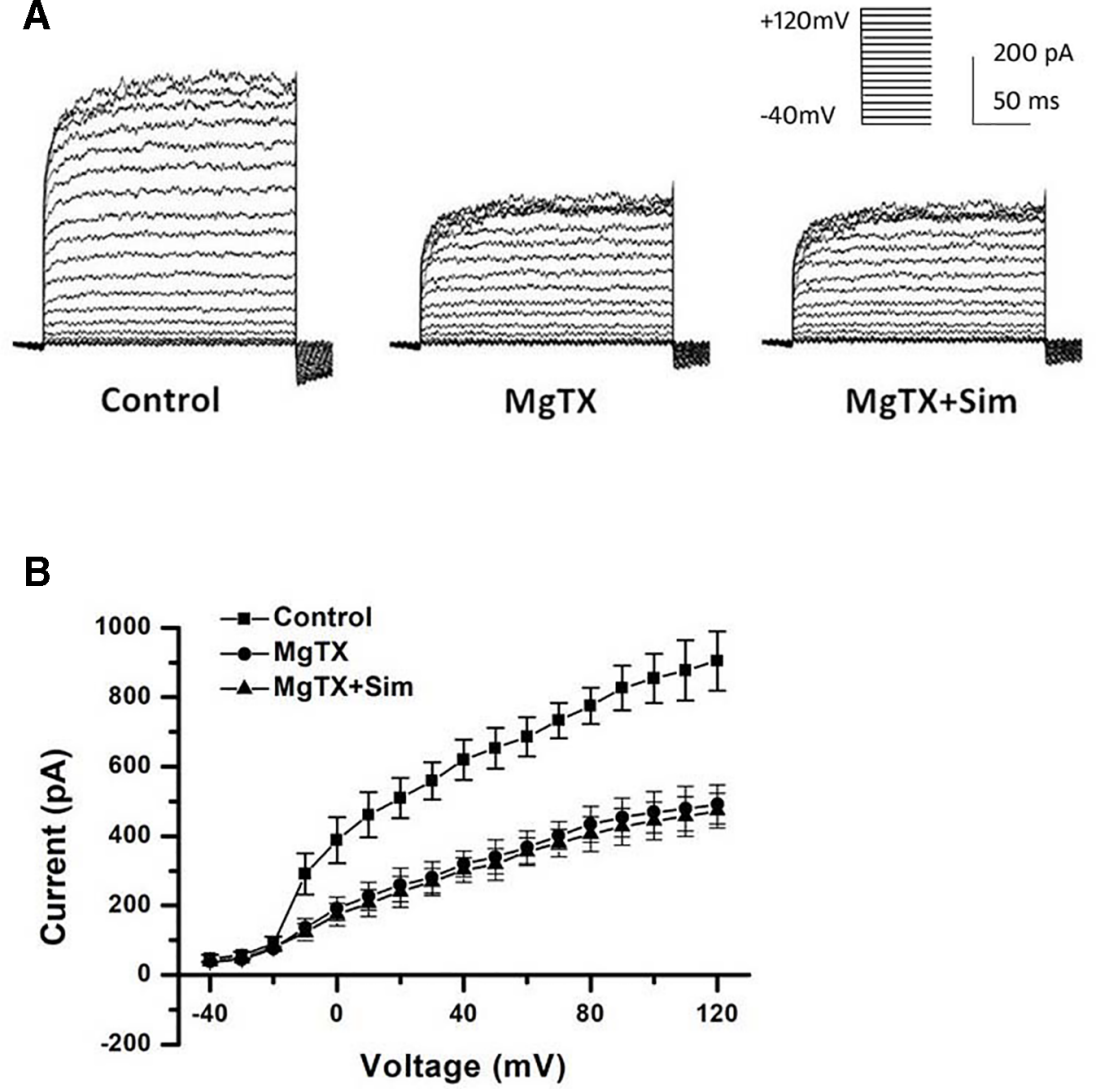
Simvastatin sensitive components of KCa3.1 current recording above −40 mV was identified in THP-1 cells measured by whole-cell clamp patch. **(A)** The THP-1 cell was held at −40mV and received the test potentials from −120 to +40 mV; the currents were detected by using an electrode with 1 µM free Ca^2+^. **(B)** Average current density-voltage relationships before and after application of 1 nM MgTX and 10 µM simvastatin was indicated (n = 4 for each group).

Besides the down-regulation of channel expression, the potential acute inhibition of functional Kv1.3 and/or KCa3.1 was evaluated by whole-cell patch clamp. The THP-1 cells were held at −70 mV, and received the test potentials from −80 to +80 mV in 10 mV steps. The specific Kv1.3 current was detected as a voltage-dependent outward potassium current. The currents were blocked by 1 nM MgTX, which is a blocker of Kv1.3 (**Figure 3A**). Simvastatin (10 μM) treatment also remarkably inhibited the current intensity of Kv1.3. The currents were further blocked by 1 nM MgTX (**Figure 3B**). When the voltage was set at +80 mV, 10 μM simvastatin significantly decreased the current intensity of Kv1.3 *vs.* control, and the addition of 1 nM MgTX further inhibited the current intensity compared with simvastatin treatment (**Figure 3C**). Using the Boltzmann equation for non-linear fitting, the IC50 of simvastatin on the current was calculated to be 8.75 ± 1.25 μM (**Figure 3D**). When the THP-1 cells were held at −40 mV and received the test potentials from −120 to +40 mV, the KCa3.1 currents were detected by using an electrode with 1 µM free Ca^2+^. The currents were blocked by 1 μM Tram-34 (**Figure 4A**). However, simvastatin (10 μM) had no effect on the current intensity of KCa3.1 as shown in **Figures 4B and C**. The slope conductance between −120 mV and −40 mV was measured by fitting the curve with linear equation. The slope conductance was 1.49 ± 0.24 nS at control, and 1.36 ± 0.05 nS with simvastatin application (n = 4, *P >* 0.05) (**Figure 4D**). Addition of 1 μM Tram-34 significantly decreased the slope conductance to 0.51 ± 0.06 nS (*P* < 0.01). To further identify the simvastatin sensitive current components above −20 mV, the THP-1 cells were held at −40 mV and received the test potentials from −40 mV to +120 mV by using the pipette solution for KCa3.1 currents recording. In the presence of 1 nM MgTX, 10 μM simvastatin had no effect on currents above −20 mV (**Figure 5**). This indicated that the currents recorded in this voltage range were contaminated by currents through the Kv1.3 channel, which was also expressed in THP-1 cells.

### Effects of Simvastatin on THP-1 Cell Migration

**Figure 6:**
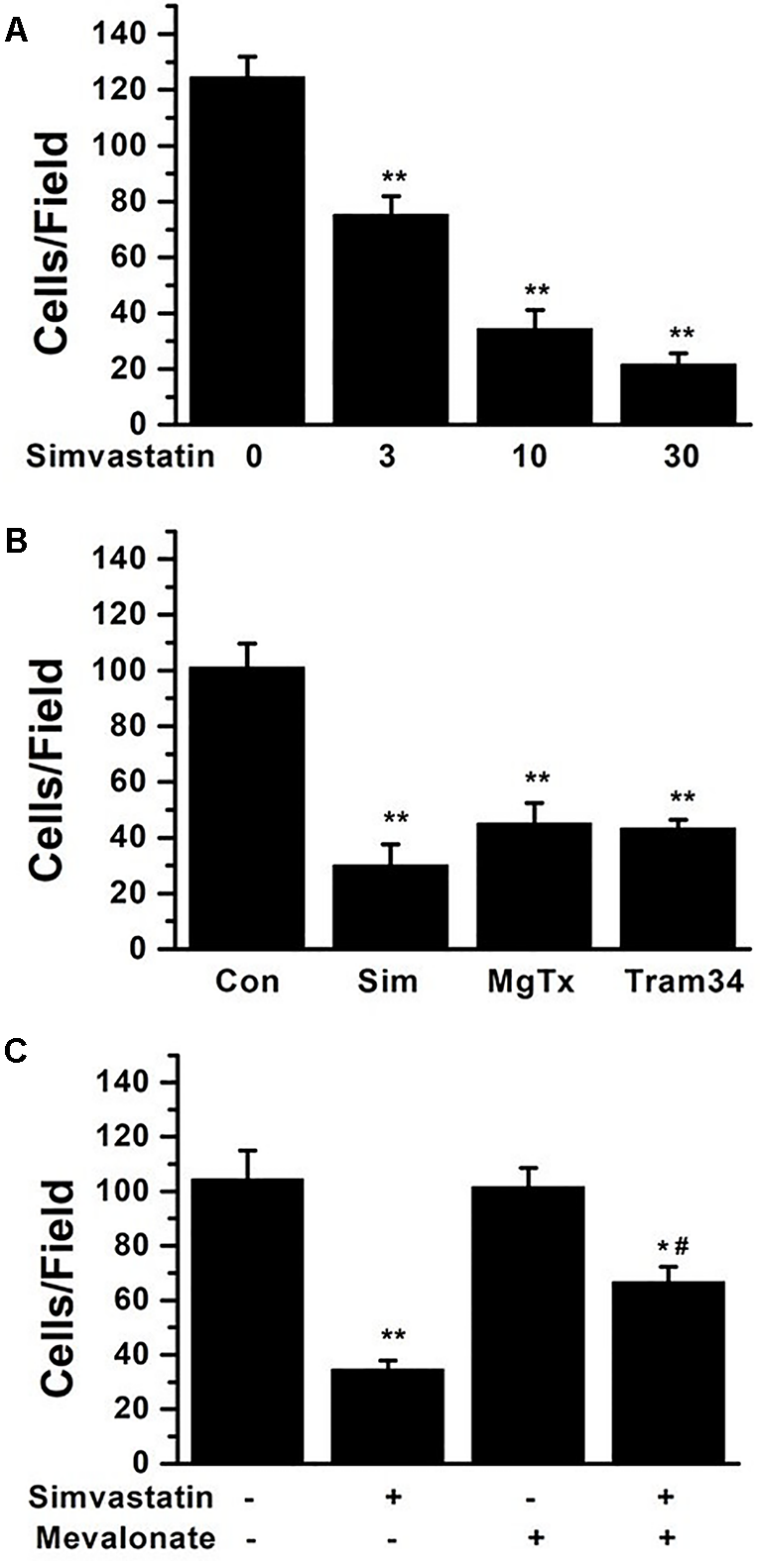
THP-1 cell migration induced by 2 nM MCP-1 was determined by immunocytochemistry in transwell cell chambers. **(A)** THP-1 cells were seeded and incubated with 0, 3, 10, or 30 µM simvastatin. The summarized data from at least 5 duplicates was expressed as mean ± SEM. **P < 0.01, *vs.* 0 µM simvastatin. **(B)** 10 µM simvastatin, 1 nM MgTX, and 1µM Tram-34 treatments significantly inhibited cell migration compared with controls, ^**^ *P* < 0.01; n = 8 for each group. **(C)** THP-1 cells were treated using 10 µM simvastatin with or without 100 μM mevalonate application, *P < 0.05 and **P < 0.01, *vs.* controls group without either simvastatin or mevalonate, ^#^ *P* < 0.05, *vs.* group with simvastatin only; n = 8 for each group.

MCP-1 (2 nM) was used as an inducer to observe monocytes migration by immuno-cytochemistry in Trans-well cell chambers. Simvastatin concentration-dependently reduced the cell migration induced by MCP-1 (**Figure 6A**). Cell migrations were also inhibited by 1 nM MgTX (45.25 ± 7.19 cells/field) and 1 μM Tram-34 (43.50 ± 2.96 cells/field) treatments (**Figure 6B**). 10 µM simvastatin (30.25 ± 7.44 cells/field) had similar effect on inhibition of cell migration compared to MgTX and Tram-34 (both *P* > 0.05). Since statin can inhibit cell migration by isoprenoid pathway (Chan et al., 2012; Bao and Zheng, 2015), 100 μM mevalonate was applied along with 10 μM simvastatin. The inhibiting effect of simvastatin on cell migration was only partially neutralized from 34.75 ± 3.15 cells/field to 66.75 ± 5.45 cells/field (*P *< 0.05) (**Figure 6C**).

### Effects of Simvastatin on the mRNA Expression of Kv1.3 and KCa3.1 in CAD Patients

**Figure 7:**
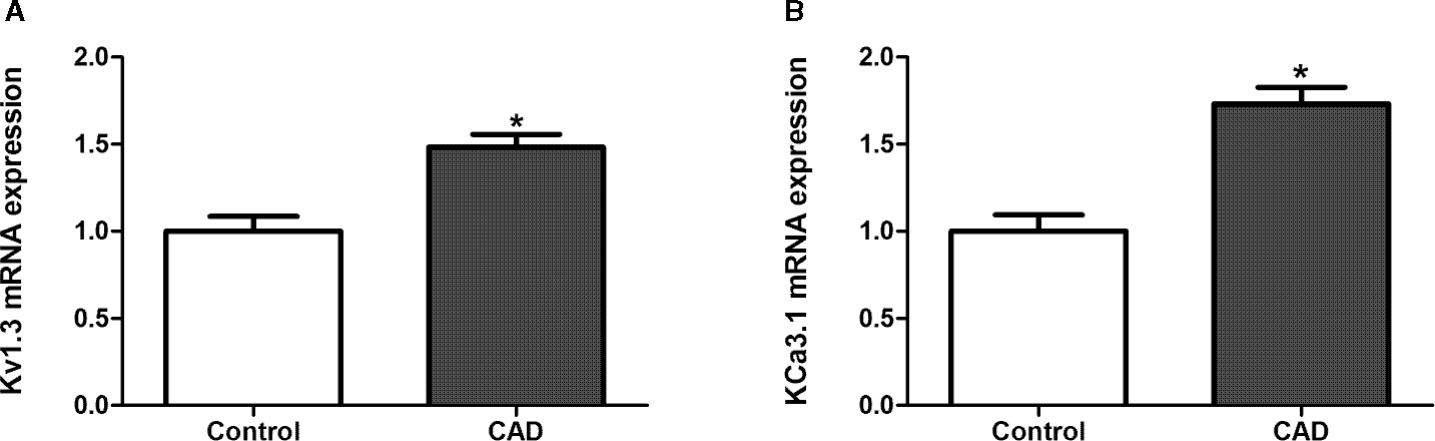
The mRNA expression of Kv1.3 and KCa3.1 in peripheral blood mononuclear cells (PBMCs) collected from healthy persons and patients with coronary atherosclerotic artery disease (CAD), respectively, determined by quantitative real-time PCR. The samples from healthy persons were used as controls. **(A)** The mRNA expression level of Kv1.3 in CAD patients (n = 20) was significantly higher than controls (n = 22), *P < 0.05. **(B)** The mRNA expression level of KCa3.1 in CAD patients (n = 20) was significantly higher than controls (n = 22).

Initially, the PBMCs from healthy controls (n = 22) and CAD patients (n = 20) were collected in Anzhen Hospital with formal permission. The mean (SEM) age was 64.20 (10.14) years old. 76.19% (n=32) was male. 66.67% (n=28) had hypertension, and 42.86% (n=18) had diabetes mellitus. The pretreatment low-density lipoprotein cholesterol was 2.48 ± 0.96 mmol/l. These baseline characteristics were comparable between healthy controls and CAD patients (All *P >*0.05). Quantitative real-time PCR was used to determine the mRNA expression levels of Kv1.3 and KCa3.1 in the PBMCs from healthy controls and CAD patients. Both Kv1.3 and KCa3.1 were expressed in PBMCs collected from the healthy controls and CAD patients. We found that the mRNA expression levels of Kv1.3 were significantly higher in the CAD patients than in the controls (**Figure 7A**). However, compared with the controls, the mRNA expression levels of KCa3.1 in CAD patients were also significantly higher (**Figure 7B**) (*P < 0.05).

**Figure 8:**
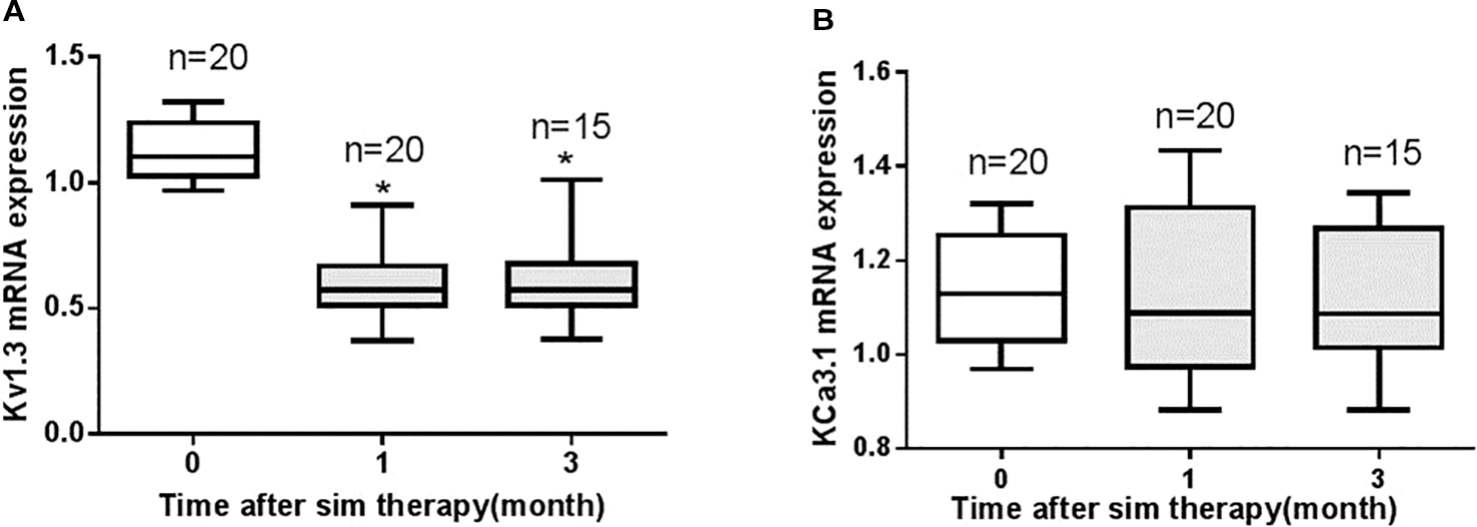
The changes in mRNA expression levels of Kv1.3 and KCa3.1 in peripheral blood mononuclear cells (PBMCs) collected from patients with coronary heart disease (CAD) prior to therapy (0 month, n=20), 1 month (n=20), and 3 months (n=15) after 40 mg per day simvastatin therapy, as measured by real-time PCR. **(A)** Simvastatin therapy significantly reduced the mRNA expression level of Kv1.3 compared with the level before simvastatin therapy (0 month), *P < 0.05; **(B)** Simvastatin had no significant effect on the mRNA expression level of KCa3.1.

All CAD patients had no history of statin therapy and received simvastatin 40 mg per day after a diagnosis of CAD. The change of mRNA expression of Kv1.3 and KCa3.1 in PBMCs were detected 1 month and 3 months after simvastatin therapy. The simvastatin therapy significantly reduced mRNA expression of Kv1.3 after 1 month and persisted to 3 months after therapy (**Figure 8A**). There was no significant difference of the mRNA expression of Kv1.3 between 1 month and 3 months after simvastatin therapy (*P* = 0.93). By contrast, the mRNA expression level of KCa3.1 showed no apparent difference after simvastatin therapy over time (**Figure 8B**). Thus, simvastatin reduced the expression of Kv1.3 mRNA, but had no influence on the mRNA expression level of KCa3.1 in CAD patients.

## Discussion

Kv1.3 and KCa3.1 currents were recorded in THP-1 cells by whole-cell patch clamp recordings. Simvastatin inhibited Kv1.3 current intensity but had no significant inhibitory effect on KCa3.1 current. The IC50 of simvastatin on the Kv1.3 current was calculated to be 8.75 ± 1.25 μM. KCa3.1 channel activity/current was determined between the voltage −120 mV to −40 mV because in this voltage range, the current recorded was purely ascribable to KCa3.1 as it was devoid of contamination by current through the Kv1.3 channel that are also expressed in THP-1 cell (Cahalan and Chandy, 2009; Chimote et al., 2013). According to our data in **Figure 4B**, simvastatin (10 μM) had no effect on the current intensity of KCa3.1 between the voltage −120 mV to −40 mV. The inhibitor effect of simvastatin on currents above −40 mV was due to the presence of currents of Kv1.3 channel. This effect was antagonized by MgTX (**Figure 5**). Although the higher concentration of simvastatin may have influence on KCa3.1, the concentration of the selected was postulated by the oral dosage used by CAD patients, and it was within the effective range and was clarified in other labs (Seto et al., 2007; Chan et al., 2012; Kazama et al., 2014; Sohn et al., 2017). Then, 10 μM simvastatin was used to investigate the effects of simvastatin on the mRNA and protein expression of Kv1.3 and KCa3.1. Simvastatin significantly inhibited the mRNA and protein expression of Kv1.3 but did not affect the expression of KCa3.1 in THP-1 cells. Simvastatin remarkably inhibited the cell migration induced by MCP-1 as well as Kv1.3 specific blocker MgTX and KCa3.1 blocker Tram-34. This study found that the mRNA expression levels of Kv1.3 and KCa3.1 in the PBMCs of patients with CAD were significantly higher than those in the PBMCs of healthy controls and simvastatin treatment inhibited the expression of Kv1.3 but not KCa3.1. These results suggest that Kv1.3 and KCa3.1 may be novel molecular markers of CAD. Simvastatin inhibits the mRNA expression and current intensity of Kv1.3 as well as the cell migration of monocytes induced by MCP-1. Kv1.3, not KCa3.1, may be the potential target of pleiotropic effect of statins providing new evidence for the clinical use of statins in the treatment of CAD. The results of the present study demonstrated the specific expression and function of ion channels expressed in non-excitable cells, especially Kv1.3 and KCa3.1 channels. Interestingly, our partial samples obtained from clinical blood samples will be more persuasive and robust to some extent.

Cholesterol in plasma membrane is known to affect many kinds of ion channels. It was reported that cholesterol is an integral component of the cell membrane, which regulates the activity of ion channels in the lipid bilayer. There are at least three potential mechanisms of influence, which includes the following: Firstly, direct interaction with the channel protein. Secondly, change in the fluidity of the bilayer by affecting ion channel gating and conformational changes. Thirdly, compartmentalization of ion channels into spatially restricted signaling complexes (called as “lipid rafts”), which merged in intestinal epithelial cells (Purcell et al., 2011). Simvastatin could change the amount of cholesterol in plasma membrane *via* indirect interaction with ion channels. In our study, western blotting, patch clamp, and cell migration experiments were introduced to clarify that channel protein expression, current change, and even cell migration coincide with the findings that the Kv1.3, not KCa3.1, was involved with the effects of simvastatin.

The ion channels expressed in non-excitable cells are closely related to the migration of tumor cells (Schwab et al., 2007), the activation of T lymphocytes (Beeton et al., 2001), and the proliferation (Wang et al., 2008a) and apoptosis (Yu et al., 1997; Wang et al., 2008b) of bone marrow stem cells. There are also many ion channels in human PBMCs that are closely related to the migration of monocytes and the secretion of inflammatory factors (Gendelman et al., 2009; Schilling and Eder, 2009; Wehrhahn et al., 2010; Wuensch et al., 2010). Monocyte ion channels may be new molecular markers and targets for drug therapy in atherosclerosis. Increased expression of potassium channels or the use of specific ion channel blockers can cause an increase or decrease in K^+^ current in whole cells, resulting in changes in downstream signaling molecules and cellular functions (Schwab et al., 2007). There is an increased expression of potassium channels Kv1.3 and KCa3.1 in PBMCs from CAD patients compared with normal healthy controls. Kv1.3 and KCa3.1 could be used as predictive molecules. Simvastatin can down-regulate Kv1.3 mRNA expression and inhibit monocyte migration *in vitro*, which is a key step of the early development of atherosclerosis. This study further verifies that simvastatin may have anti-atherosclerotic effect by targeting and regulating Kv1.3 potassium channels. The possible mechanisms for the pleiotropic effects of statins could be closely related to potassium channels. However, both mRNA and protein of KC3.1 had no significant change as well as the current density after treatment with simvastatin. This finding showed that KCa3.1 was unlikely to be the target of simvastatin. The increase in KCa3.1 mRNA expression level in PBMCs from CAD patients compared with healthy controls was related to other reflections of atherosclerosis and regulated by other pathways. Inhibition of KCa3.1 by Tram-34 decreased monocyte migration, which indicated the significance of KCa3.1 in the development of atherosclerosis. Toyama K et al (Toyama et al., 2008) found that macrophages, visualized in lesions by immunostaining for Mac3, also expressed KCa3.1 in plaques of apoE-/- atherosclerosis mice aortic sinus. Blockade of KCa3.1 with TRAM-34 also partially reduced migration of MCP-1- and lysophosphatidylcholine-stimulated monocytes (Schilling and Eder, 2009). Our data were consistent with these findings.

Despite the biological effects of statins on functional expression of Kv1.3 and migration of monocytes, the current study does not identify the confirmable mechanism of association between monocyte migration and Kv1.3 function that was regulated by statins. Mevalonate pathway was reported to be involved the migration of smooth muscle cells, which was attenuated by simvastatin (Chan et al., 2012) and atorvastatin (Bao and Zheng, 2015). However, in our study, mevalonate could only partially neutralize the inhibiting effect of simvastatin on monocyte migration. Extracellular signal-regulated kinase (ERK) is one of the major classes of mitogen-activated protein kinases (MAPK), which regulate a wide array of cellular processes (Whelan et al., 2012). ERK activity is the downstream signal of Kv1.3, which mediated the macrophage migration (Kan et al., 2016). Further studies such as Kv1.3 knockdown (Zhao et al., 2015) are required to demonstrate the molecular mechanisms that mediated simvastatin and/or MgTx induced inhibition of monocyte migration. Mevalonate is one of the blockers in isoprenoid pathway (Chan et al., 2012). Our data showed that mevalonate could only partially the neutralize effect of simvastatin on cell migration. This indicated that ion channel inhibition could be another potential pathway.

After simvastatin treatment, the mRNA expression level of Kv1.3 in PBMCs of CAD patients decreased significantly compared to the level of pretreatment. This effect persisted at least for 3 months. However, mRNA expression of KCa3.1 in PMBCs did not decrease after 3 months of anti-atherosclerotic therapy. It will be interesting to investigate the association between the dynamic change of Kv1.3 mRNA in PBMCs and atherosclerosis progression or regression. We will attempt to supplement these results through animal studies in future experiments. Moreover, in our study, we investigated the effects of simvastatin only. Whether other statins with different lipophilicity have similar anti-inflammatory effect needs to be clarified further.

Statins may modulate potassium channel activity in other tissues, such as skeletal muscle, where they may induce side effects. An increase of the total potassium conductance has been found in skeletal muscle of rats treated with 10 mg/kg and 50 mg/kg simvastatin. This increase was restored by the *in vitro* application of glybenclamide, a specific blocker of the ATP-sensitive potassium channels (Pierno et al., 1995; Pierno et al., 2006), suggesting their involvement and that these channels are activated to recover the ATP reduction. In contrast, atorvastatin did not modify the total gK in skeletal muscle of chronically treated rats (Camerino et al., 2016).

It has become the frontier of atherosclerosis research to study mononuclear cell biology behavioral changes during the early stages of atherosclerosis formation to identify the key signaling molecule regulating cell-induced atherosclerotic changes and, thus, a new target that is closely related to the development and progression of atherosclerosis. Clinical samples, including PBMCs, collected before and after simvastatin therapy from CAD patients were employed in this study, which provided strong evidence and support for basic investigations using THP-1 cells. However, there are still some deficiencies regarding *in vivo* study. Several *in vivo* knockout animal studies and *in vitro* human tissue sample studies would further support this hypothesis.

## Ethics Statement

All applicable international, national, and/or institutional guidelines for the care and use of animals were followed. All procedures performed in studies involving human participants were in accordance with the ethical standards of the institutional and/or national research committee and with the 1964 Helsinki declaration and its later amendments or comparable ethical standards. Informed consent was obtained from all individual participants included in the study.

## Author Contributions

SW and YR performed the research design, data analysis, and writing of the manuscript. Both of them are the cofirst authors. XC performed the molecular biochemistry tests and CL completed the patch clamp experiments. SC performed the clinical sample collection and preparation. JL contributed to research design and manuscript revision.

## Funding

This study was funded by the Beijing Natural Science Foundation (grant number: 7122058), the National Basic Research Program of China (973 Program, 2015CB554404), and the Beijing Lab for Cardiovascular Precision Medicine, Beijing, China (PXM2016_014226_000023).

## Conflict of Interest

The authors declare that the research was conducted in the absence of any commercial or financial relationships that could be construed as a potential conflict of interest.
